# A New Extraction Method of Loess Shoulder-Line Based on Marr-Hildreth Operator and Terrain Mask

**DOI:** 10.1371/journal.pone.0123804

**Published:** 2015-04-24

**Authors:** Sheng Jiang, Guoan Tang, Kai Liu

**Affiliations:** 1 Key Laboratory of Virtual Geographic Environment, Ministry of Education, Nanjing Normal University, 1 Wenyuan Road, Nanjing, Jiangsu 210023, China; 2 State Key Laboratory Cultivation Base of Geographical Environment Evolution (Jiangsu Province), 1 Wenyuan Road, Nanjing, Jiangsu, 210023, China; 3 Jiangsu Center for Collaborative Innovation in Geographical Information Resource Development and Application, 1 Wenyuan Road, Nanjing, Jiangsu 210023, China; Cardiff University, UNITED KINGDOM

## Abstract

Loess shoulder-lines are significant structural lines which divide the complicated loess landform into loess interfluves and gully-slope lands. Existing extraction algorithms for shoulder-lines mainly are based on local maximum of terrain features. These algorithms are sensitive to noise for complicated loess surface and the extraction parameters are difficult to be determined, making the extraction results usually inaccurate. This paper presents a new extraction approach for loess shoulder-lines, in which Marr-Hildreth edge operator is employed to construct initial shoulder-lines. Then the terrain mask for confining the boundary of shoulder-lines is proposed based on slope degree classification and morphology methods, avoiding interference from non-valley area and modify the initial loess shoulder-lines. A case study is conducted in Yijun located in the northern Shanxi Loess Plateau of China. The Digital Elevation Models with a grid size of 5 m is applied as original data. To obtain optimal scale parameters, the Euclidean Distance Offset Percentages between shoulder-lines is calculated by the Marr-Hildreth operator and the manual delineations. The experimental results show that the new method could achieve the highest extraction accuracy when σ = 5 in Gaussian smoothing. According to the accuracy assessment, the average extraction accuracy is about 88.5%, which indicates that the proposed method is applicable for the extraction of loess shoulder-lines in the loess hilly and gully areas.

## Introduction

The Loess Plateau of China is famous for its unique erosion landscape features of thick loess accumulation and numerous valleys. Many researches have been carried out in the region over the last half a century, including soil erosion [1–3], land evaluation[4,5], topographic features analysis[6–8], etc. In many features which characterize the loess morphological landform, loess shoulder-lines are regarded as vital structural lines which divide the complicated loess valley into loess interfluves and gully-slope lands. The loess shoulder-lines directly reflect the staggered distribution characteristics of loess positive and negative terrain. It also reveals the model of evolution of soil erosion. Thus, the loess shoulder-line is an effective research point for spatial form distribution and geomorphic evolution of loess landform.

The extraction of loess shoulder-lines is a significant and challenging research work for the peculiar and complicated loess physiognomy. In the 1990s, the loess shoulder-lines are delineated manually on the topography maps and remote sensing images by interpreters, which is a time-consuming and subjective way. In recent years, with the technological advance in satellite sensors and GIS spatial analysis, the algorithms for loess shoulder-lines extraction have been studied based on Digital Elevation Model (DEM) and high resolution remote sensing images. For example, Lu[9] proposed a morphological method by utilising the finite morphologic elements to define the topographical structure. Specifically, loess shoulder points are extracted using eight neighbour windows and slope variability. Then, these points are connected into lines by recursive search. The major limitation of the interpolation method results from noise-sensitive in complex terrain. Zhu[10] extracted the loess shoulder-lines by three features: lope variability, profile curvature and channel network. The experiment results showed that the multi-feature extraction method could improve the extraction stability compared with the single feature methods. However, further study is needed for improving the efficiency and effectiveness in features fusion. Liu[11] found that the buffer of the flow path can be used to simplify valley profiles and increase extraction accuracy. According to the forms of loess slope characteristics, Zhou[12] and Song[13] adopt snake model to gradually reach the loess shoulder-lines in order to overcome the problems of lines discontinuity and improve the accuracy. The limitation of the snake extraction method is that it is not efficient and needs to set the iteration numbers by researchers. Li[14] took efficient LoG operator for DEM edge detection. But, it does not solve the parameter setting problem in loess broken valleys. From the comparative analysis of previous researches, there are several problems which require further study: (1) The extraction of shoulder points is the key step in slope variability based methods and the existing line connecting algorithm. The regular line connecting algorithm cannot compensate for the precision loss in points’ extraction. (2) The existing algorithms are sensitive to noise especially in the area with complicated land surface and the extraction parameters are difficult to be determined, making the extraction results usually inaccurate. (3) Existing researches is more dependent on the single data source, i.e. DEM data and multi-source data and auxiliary data have not utilized to improve extraction accuracy. Hence, to obtain an efficient algorithm it is not only necessary to extract the loess shoulder-lines from the formation mechanism and morphologic features, but also needs to consider the processes and methods. In addition, reasonable auxiliary data is an important way to improve data accuracy.

This paper presents a new extraction approach for loess shoulder-lines, by which Marr-Hildreth edge operator is employed to construct initial shoulder-lines. Then a new terrain mask used for confining the boundary of shoulder-lines is proposed based on slope degree classification and morphology methods in order to avoid interference from non-valley area and modify the initial loess shoulder-lines. Terrain mask is obtained by global threshold classification. The optimal parameter of Marr-Hildreth operator is calculated by Euclidean Distance Offset Percentages. A case study is conducted in Yijun which is located in the northern Shanxi Loess Plateau of China. At last, some more examples are provided to show the effect and accuracy of this proposed new method compared with published extraction method.

Specific objectives included: (1) a simple method for terrain mask; (2) a process for extracting shoulder-lines with constraint of terrain masks; (3) parameter analysis and selection of Marr-Hildreth operator and accuracy assessment.

## Study Area

### 2.1 Ethics statement

No specific permits were required for the described studies. The work did not involve any endangered or protected species.

### 2.2 Study area and data

**Fig 1:**
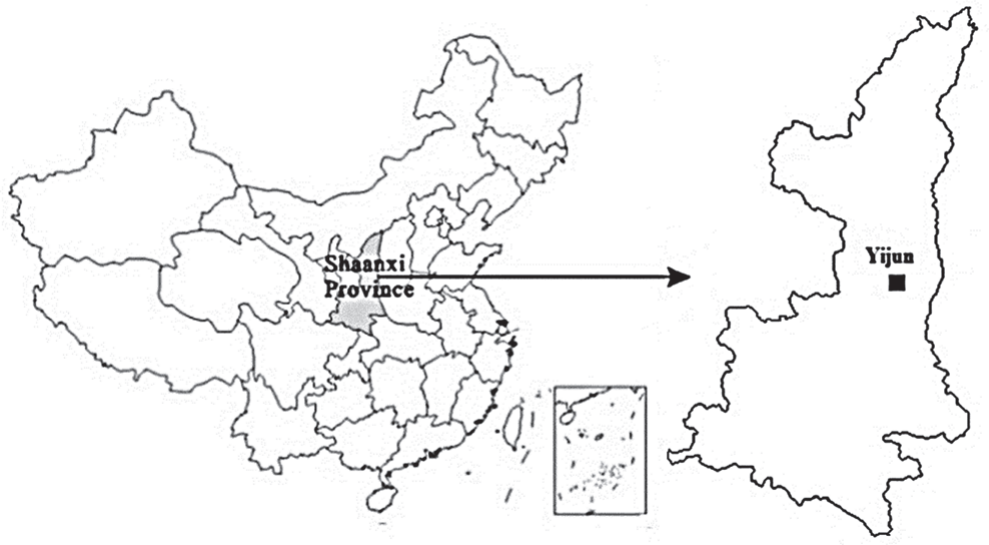
The study area is located in Yijun, Shanxi province of China.

The study area is Yijun that situated in the middle of the Loess Plateau, Shanxi province, China (Fig 1). Yijun is the loess residual tableland area and centred on 35°25’22”N, 109°38’52”E. The reasons that Yijun was chosen as the study area were, firstly, the typical landform type of Yijun is loess hilly and gully area where typical loess shoulder-lines could be extracted; secondly, the latest high resolution DEM data has been obtained, as well as the high spatial resolution DOM which could be used to verify the accuracy of loess shoulder-lines. The elevation is from 1125 m to 1742 m height. The gully density is 4.2 km/km^2^. This landform is the residual state of loess tableland and in the later geologic development stage of the landform. Although with a flat top surface with similar height, the overall tableland surface is actually dissevered into separated blocks. The shoulder-lines are in petaloid and finger like structure. The DEM data of Yijun in this research is from the contours of 1:10000 topographical maps and produced by the National Geomantic Centre of China with a spatial resolution of 5 m.

## Methods

### 3.1 Marr-Hildreth operator

**Fig 2:**
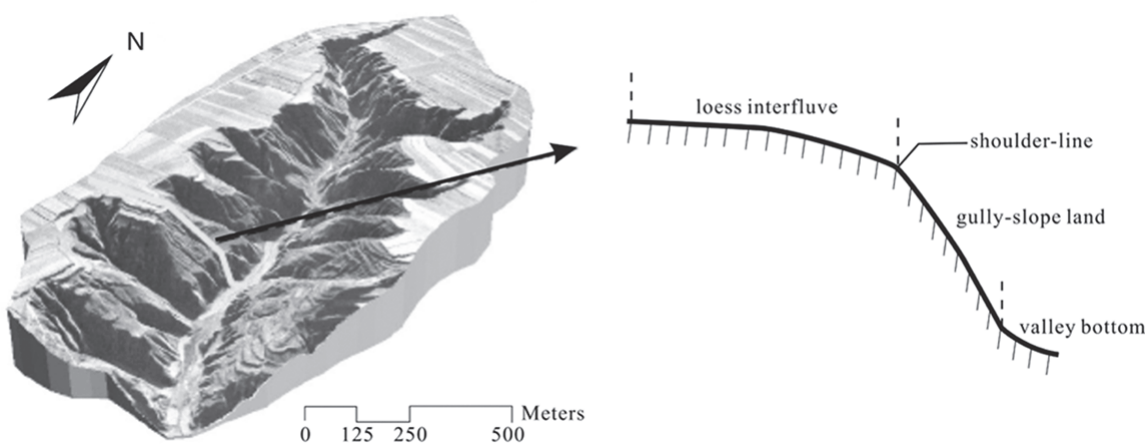
A shoulder-line’s location in a typical profile.

The valley region in Loess Plateau is of obvious morphological characteristics. They are composed of loess interfluves, gully-slope lands and valley bottom lands[3]. Among them, loess shoulder-lines are the dividing lines between smooth loess interfluves and steep gully-slope lands. There are significant discontinuous turning points between gully-slope land and loess interfluve. As shown in Fig 2, a higher value of the second-order derivatives could be found where the loess shoulder-lines are located[9]. Edge detection is an important image processing technique for finding the boundaries of objects within images. It works by detecting discontinuities in image values. Meanwhile, a DEM is a grid cell-based dataset that could be regarded as a specific image[15]. The common detection approach is also suitable for DEM based structural lines extraction. As a result, this paper use edge detection technique to extract the loess shoulder-lines.

The edge detection methods can be divided into two categories, namely, zero-crossing based and search-based[16]. Specifically, common edge detection algorithms include Sobel, Canny, Prewitt, Roberts, and Marr-Hildreth operator. Due to lots of small terrain objects and terraced fields in the complicated loess surface, the edge detection operator need to efficient and anti-noise. Canny operator is complicated in parameterization and it is time-consuming. Sobel, Prewitt, et al. is sensitive to noise. therefore the Marr-Hildreth operator is selected for automatic extraction of the loess shoulder-lines[14]. The Marr-Hildreth operator is based on the detection of zero-crossings of the Laplacian operator applied to a Gaussian-smoothed image[17]. The pre-process, Gaussian smoothing, is implemented as an initial step of the procedure. Then, the dual edges are extracted by Laplacian operator and the final edges are located by zero-crossing method.

Gaussian smoothing function is defined as:

$$G_{\sigma }(x,y)=\frac{1}{\sqrt{2\pi \sigma^{2}}}e^{-(x^{2}+y^{2})/2\sigma^{2}}$$ 1

where *σ* denotes the standard deviation value of elevation in a moving window, and the *x* and *y* indicates the location of the elevation grid. In general, a great *σ* is indicative of a more obvious smoothing effect.

The first order and the second order derivative of *x* are defined as Eq (2) and Eq (3):

$$\frac{\partial }{\partial x}G_{\sigma }(x,y)=\frac{\partial }{\partial x}e^{-(x^{2}+y^{2})/2\sigma^{2}}=-\frac{x}{\sigma^{2}}e^{-(x^{2}+y^{2})/2\sigma^{2}}$$ 2

$$\frac{\partial^{2}}{\partial^{2}x}G_{\sigma }(x,y)=\frac{x^{2}}{\sigma^{4}}e^{-(x^{2}+y^{2})/2\sigma^{2}}-\frac{1}{\sigma^{2}}e^{-(x^{2}+y^{2})/2\sigma^{2}}=\frac{x^{2}-\sigma^{2}}{\sigma^{4}}e^{-(x^{2}+y^{2})/2\sigma^{2}}$$ 3

Similarly, the second-order derivative of y is expressed as the Eq (4):

$$\frac{\partial^{2}}{\partial^{2}y}G_{\sigma }(x,y)=\frac{y^{2}-\sigma^{2}}{\sigma^{4}}e^{-(x^{2}+y^{2})/2\sigma^{2}}$$ 4

Finally, the specific form of LoG operator is defined as:

$$LoG(x,y)=\frac{\partial^{2}}{\partial^{2}x}G_{\sigma }(x,y)+\frac{\partial^{2}}{\partial^{2}y}G_{\sigma }(x,y)=\frac{x^{2}+y^{2}-2\sigma^{2}}{\sigma^{4}}e^{-(x^{2}+y^{2})/2\sigma^{2}}$$ 5

In summary, this algorithm uses Gaussian operator to smooth the DEM data, then the dual edges are extracted by Laplacian operator and the final edges are located by zero-crossing method.

### 3.2 Terrain mask

So far, there have been many previous and systematical studies on the factors affecting the extraction of loess shoulder-lines, and provide three main factors which interference the efficiency and effectiveness of loess shoulder-lines extraction[9,11,13,18]: (1) Terrain fields’ interference. There is obvious slope transition in the border area between terraces. The extension direction of this transition is nearly the same as the development direction of loess gully. (2) Smooth slope. Since severe soil erosion and landslides, the border slope areas between loess interfluves and gully-slope lands probably show a smooth state, which may interference the determination of loess shoulder-lines extraction. (3) Discontinuity of shoulder-lines. Both sides of the main valley often show discontinuous stretched shoulder-lines, while the shoulder-lines of minor gulley is more clear and distinguished. These shoulder-lines within discontinuous areas need modified by connecting algorithm.

**Fig 3:**
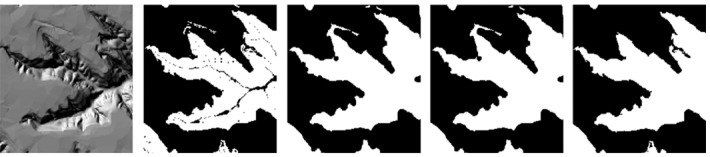
A terrain mask by different morphological method. (a) hillshade image of DEM; (b) terrain mask by Jenks natural breaks only; (c) mask after opening operation; (d) mask after closing operation; (e) mask after reconstruction.

In order to overcome the problem of local discontinuity of loess shoulder-lines, the global threshold or morphological features are employed. In addition, how to avoid interference from non-valley area is also a practical issue. Therefore, a terrain mask used for confining the boundary of shoulder-lines is proposed based on slope degree classification and morphology methods. Slope degree classification by Jenks natural breaks[19] method could achieve a reasonable result, dividing the loess valleys into positive and negative surface globally. Jenks natural breaks method can obtain the optimal arrangement of values which seeks to reduce the variance within classes and to maximize the variance between classes. Then the boundaries of terrain masks is able to modify the discontinuous loess shoulder-lines. Fig 3(A) shows the hillshade image of DEM data by ArcGIS 9.3. Fig 3(B) illustrate the slope degree classification result by Jenks natural breaks. There are some small areas and gaps. As shown in the Fig 3(C) and the Fig 3(D), the common opening or closing operation cannot get satisfactory results. Thus opening and closing by reconstruction methods are applied. Firstly, 10 pixels radius round shape *s* is utilized for eroding operation to the data *f*:

$$f_{e}=f⊖s$$ 6

The isolated objects whose area is smaller than shape *s* will be removed after eroding operation, while the objects whose area is greater than shape *s* will also shrink. Therefore, the reconstruction method[20] which original *f* is taken as the mark image is performed to the data *f*_*e*_:

$$f_{er}=R_{f}(f_{e})$$ 7

After the reconstruction operation, the objects shrank resume the original shapes. In order to fuse the pits and gaps of the objects, closing-by-reconstruction is conduct on *f*_*er*_. Using the image complement method, the dilating operation could be converted to eroding operation and it is of benefit to method coherence. The expression is as follows:

$$f_{erc}=complement(f_{er})$$ 8

At last, reconstruction operation is carried out between *f*_*erc*_ and the data eroded which is set as the mark image and the final data *f*_*erce*_ should be complemented:

$$f_{erce}=complement(R_{f_{erc}}(f_{erc}⊖s))$$ 9

The result (Fig 3E) shows that the pits and gaps in the objects whose areas is smaller than shape *s* will be fused, meanwhile the objects’ shape retains the same with original one. The extraction for terrain mask will be applied to constrain the boundary of loess shoulder-lines.

## Results and Discussion

### 4.1 Extraction of loess shoulder-lines

**Fig 4:**
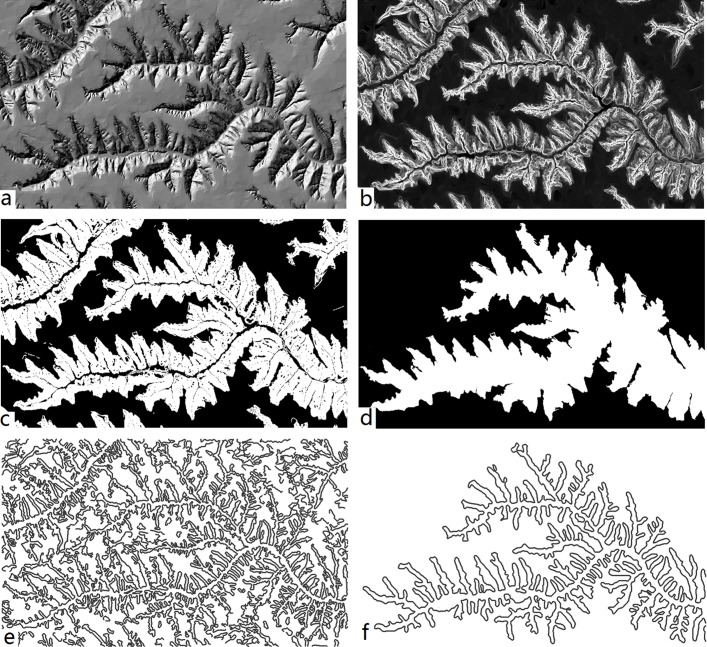
Extraction procedure of loess shoulder-lines. (a) Hillshade image of DEM data; (b) Slope image of the test area; (c) Initial terrain masks using Jenks natural breaks only; (d) The largest and main terrain mask by reconstruction operations; (e) Initial extraction result by Marr-Hildreth without terrain mask; (f) Final loess shoulder-lines with terrain mask(σ = 8).

To demonstrate the procedure and result of the extraction method proposed, a test region is selected and the extraction experiment is performed. As shown in Fig 4(A), this region is located in Yijun and it is the landform type of loess residual tableland. First of all, the slope degree image is calculated (Fig 4B), in which the legend of slope are lighter in the steep areas while darker in relative flat areas. Then the initial terrain mask is acquired based on the slope degree classification with the threshold values obtained via Jenks natural breaks (Fig 4C). In order to get complete and smooth terrain masks, opening and closing operations by reconstruction is applied to the initial terrain masks. Meanwhile, the main terrain mask is selected by largest area in the initial masks. The result image shown in Fig 4(D) indicates that the operation can filter out trivial interferential factors and fuse the small interior pits.

The loess shoulder-lines could be extracted with the masks by previous step. The Fig 4(E) shows the direct extraction result on the original DEM data by means of Marr-Hildreth operator. Obviously, some non-shoulder-line edges are also detected, which could be regarded as meaningless noise in loess shoulder-lines extraction. In addition, many small and broken edge lines are distributed in the main valley which are need to be repair and remove. The final result is shown in Fig 4(F) by the parameter σ = 8. The assistance of the terrain mask indicates that the non-shoulder-line noises have been filtered out by overlay analysis using the main terrain mask. Moreover, the eight direction using the Lu’s algorithm[9] of 5×5 window neighbourhood search is employed to connect the discontinuous lines and retains the longest lines as the main and final shoulder-lines. Because the non-shoulder-lines have been cleared, the connection process is very efficient. Consequently, terrain masks improve the extraction accuracy and efficiency of loess shoulder-lines.

### 4.2 Parameter analysis

**Fig 5:**
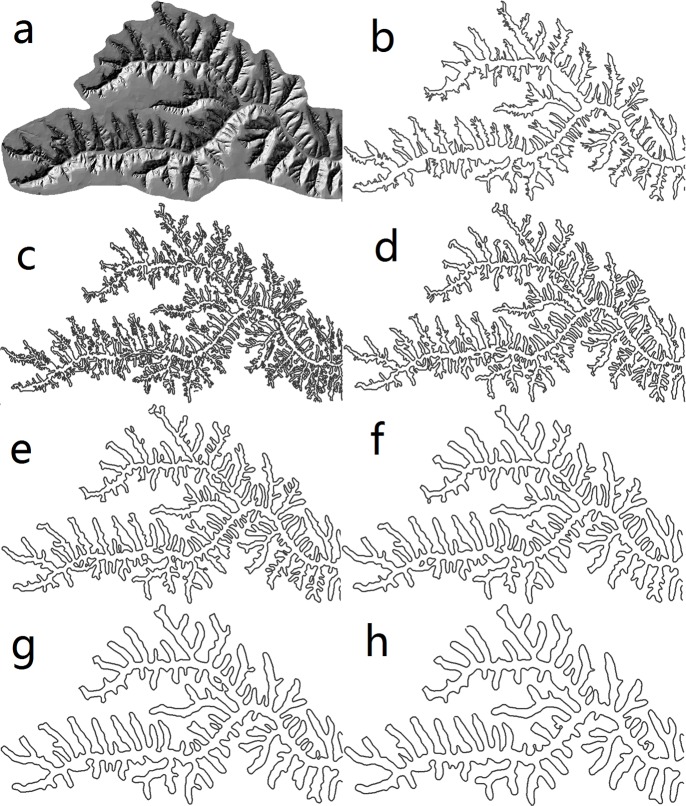
Loess shoulder-lines with different standard deviation σ. (a) Hillshade image of original DEM; (b) manual delineation; (c), (d), (e), (f), (g) and (h) are the result by different σ of 2, 4, 6, 8, 10 and 12 respectively.

The standard deviation *σ* in the Marr-Hildreth operator is usually set as the scale parameter in multi-resolution segmentation approach[21]. In this extraction method, the *σ* greatly impacts the extraction scales and shapes of loess shoulder-lines. As shown in Fig 5C–5H, these shoulder-lines are extracted with different *σ* value of 2, 4, 6, 8, 10 and 12 respectively.

In order to quantify the extraction accuracy and get the optimal parameter of the Marr-Hildreth operator, the Euclidean Distance Offset Percentage (EDOP) which is modified by the accuracy assessment method of Euclidean distance offset method[13], is proposed. The manually extraction products (Fig 5B) delineated with high-resolution digital orthophoto graphs are employed as the criterion which will assess the extraction accuracy. The first step is to calculate Euclidean distance raster layer (EDRL) with 20 m in ArcGIS 9.3 based on the manual delineation. In this paper, 20 m is offset distance threshold. In the previous research, researchers have achieved good results by the threshold of 20 m in the similar study area and the same data[12,13]. Therefore, 20 m is also used as the offset distance in this paper. Then the shoulder-lines with different *σ* values are intersected with the EDRL. If the test pixel is located in the buffer of EDRL, the pixel is considered as a relative correct one of the shoulder-lines. At last, the extraction accuracy is represented by the statistic proportion of the correct pixels in the total pixels, namely, the EDOP accuracy. Typically, the higher the EDOP, the better the accuracy of the loess shoulder-lines extraction. The relationship between *σ* and EDOP accuracy is shown in Table 1. When *σ* = 5, a highest EDOP accuracy is achieved, i.e. 89.77%.

**Table 1:** The EDOP accuracy with different standard deviation *σ*.

	*σ* = 1	*σ* = 2	*σ* = 3	*σ* = 4	*σ* = 5	*σ* = 6	*σ* = 7	*σ* = 8	*σ* = 9	*σ* = 10	*σ* = 11	*σ* = 12
LS (m)	483.77	266.37	199.41	172.97	152.45	134.87	123.24	113.81	107.42	100.47	94.84	89.26
LS within EDRL(m)	300.34	199.62	169.96	152.50	136.85	120.41	104.93	92.60	83.50	73.74	66.52	58.17
EDOP accuracy	62.08%	74.94%	85.23%	88.16%	89.77%	89.27%	85.14%	81.36%	77.74%	73.39%	70.14%	65.17%

LS: Length of shoulder-lines (m); LS within EDRL: Length of shoulder-lines within EDRL.

**Fig 6:**
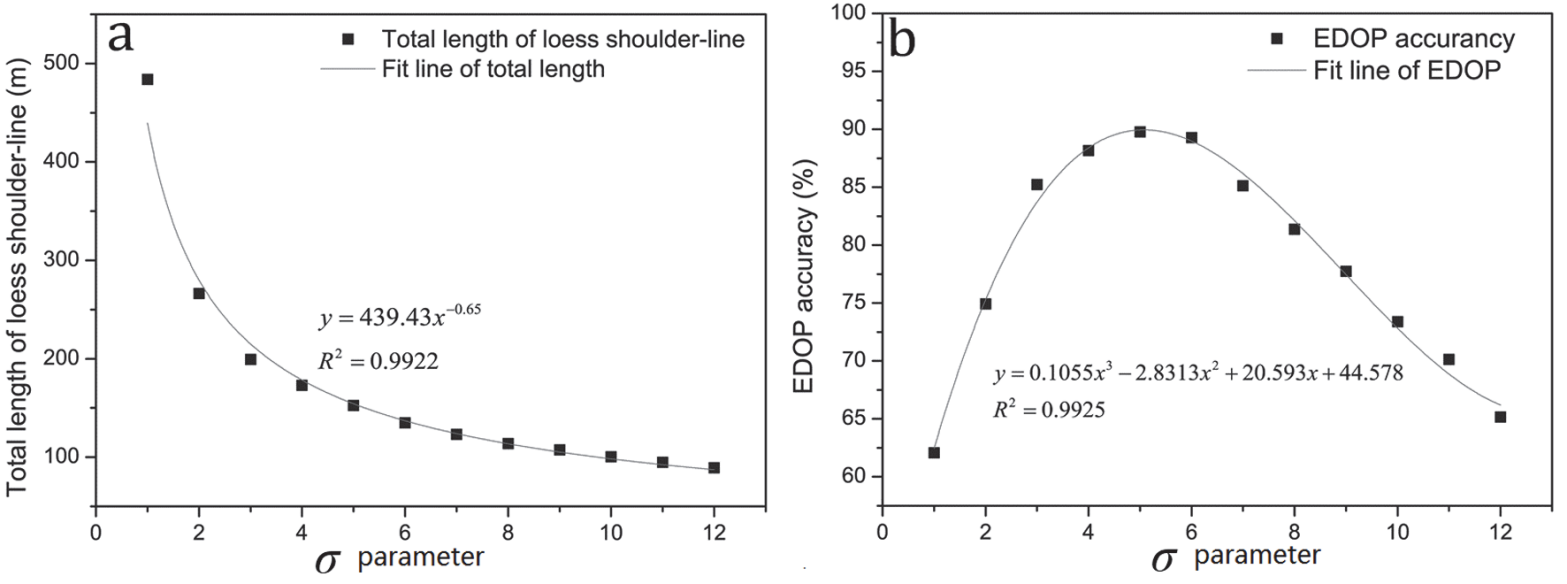
The scatter plots represent the relationship between (a) standard deviation and length of loess shoulder-lines; (b) standard deviations and EDOP accuracy.

Fig 6(A) is the scatter plot between *σ* values and the length of shoulder-lines. With the decrease of *σ* parameter, the length of shoulder-lines is increased. The regression equation is as follows:

$$y=439.43x^{-0.65}$$ 10

The relationship result between *σ* parameter values and EDOP accuracy is shown in the Fig 6(B). It is apparent that the accuracy reaches the peak while *σ* is getting 5. And the regression equation is expressed as:

$$y=0.1055x^{3}-2.8313x^{2}+20.593x+44.578$$ 11

### 4.3 Accuracy assessment

**Fig 7:**
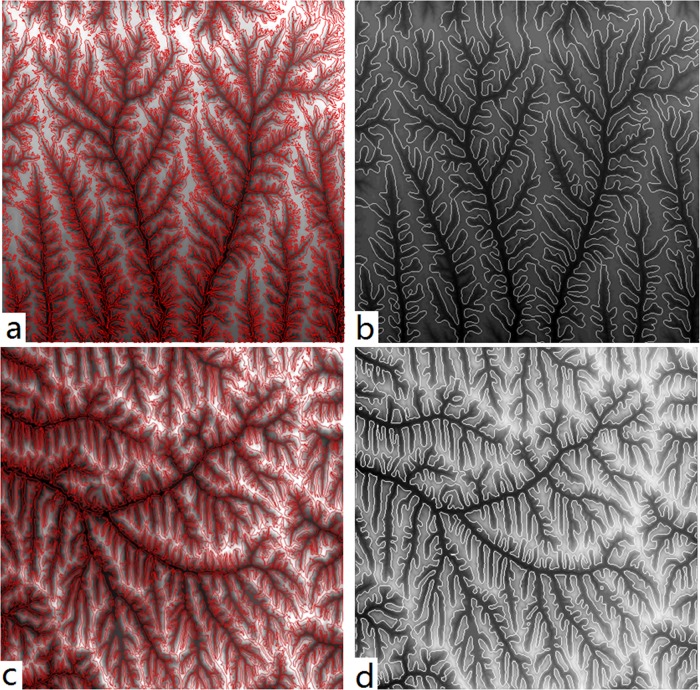
Extraction results of loess shoulder-lines obtained by (a) the Tang method[22]; (b) proposed method with σ = 5; (c) the Tang method[22]; (d) proposed method with σ = 5.

Some more examples are used to test the accuracy of this new method. According to the parameter analysis of previous section, the extraction parameter *σ* is set to 5. Fig 7 are the extraction results by our method and Tang’s method[22] in the loess gully and hilly region. Tang’s method is a simple and effective approach by neighbourhood analysis with moving windows. Fig 7(A) and 7(C) is the extraction results by the Tang’s method. Fig 7(B) and 7(D) is the results by proposed method in this paper. From the direct sense perception, Tang’s loess shoulder-lines are complicated and dense, while the proposed method provides much more clear and explicit results for the sake of smoothing operator and terrain masks. In order to verify and quantify the extraction accuracy of these two methods, the EDOP is adopted. The manually extracted products delineated with high-resolution digital orthophoto map are employed as the criterion which will assess the extraction accuracy. At last, the final assessment results are shown in Table 2. According to the accuracy assessment, the average extraction accuracy of proposed method is about 88.5%, it is better than the Tang’s method’s average accuracy, 81.2%. Moreover, the total length of shoulder-lines by proposed method is much less than Tang’s, illustrating the effectiveness of the smoothing algorithm and the terrain mask. From the accuracy analysis, the results show that the proposed method not only possesses satisfy extraction accuracy, but also can promote the geomorphometry study in the loess area.

**Table 2:** Accuracy assessment.

	Tang method		Proposed	
	Fig 7(A)	Fig 7(C)	Fig 7(B)	Fig 7(D)
Total length (km)	463.1	598.8	320.4	454.6
Effective Length within EDRL (km)	254.7	344.9	277.6	384.9
EDOP accuracy	80.1%	82.3%	87.3%	89.7%

EDRL: Euclidean distance raster layer.

## Conclusions and Future Work

This paper presents a new extraction approach for loess shoulder-lines based on Marr-Hildreth edge operator and terrain masks. Marr-Hildreth operator is employed to construct initial loess shoulder-lines. Then a novel terrain mask, confining the boundary of shoulder-lines, is also proposed based on slope degree classification and morphology methods. The experimental results show that the terrain mask can filter out the non-shoulder-line noises. Consequently, terrain masks improve the extraction accuracy and efficiency of loess shoulder-lines. Moreover, parameter analysis shows that the highest extraction accuracy of shoulder-lines could achieve when σ = 5 in Gaussian smoothing. Meanwhile, according to the accuracy assessment, the average extraction accuracy of proposed method in this paper is better than Tang’s method. In addition, the total length and shape of the shoulder-lines extracted by our method are also much more reasonable and accurate.

This research focuses on the extraction of should-lines in Yijun, representing residual state of loess tableland. Therefore, further researches are expected to explore the applicability of our method in more different types of loess landform. In addition, the formation and development mechanism of loess shoulder-lines are also expected to be investigated.
